# Wheelchair Tai Chi Ball Exercise for Improving Neuromuscular Functions of Older Adults With Disability

**DOI:** 10.3389/fnagi.2022.935986

**Published:** 2022-07-19

**Authors:** Ting Liao, Xiong-Wen Ke, Yong Tai Wang

**Affiliations:** ^1^Aquatic Therapy and Fitness Research Center, Wuhan Sports University, Wuhan, China; ^2^Department of Marital Art, Wuhan Sports University, Wuhan, China; ^3^Department of Medical Sciences, Health and Management, Rochester Institute of Technology, Rochester, NY, United States

**Keywords:** Wheelchair Tai Chi ball, mind-body exercise, strength training, neuromuscular functions, older adults, disability

## Abstract

The purposes of this perspective article were to summarize Wheelchair or Seated Tai Chi studies related to neuromuscular functions of older adults with disability; to describe the development of Wheelchair Tai Chi Ball (WTCB) exercise — a concept to combine mind-body exercise with strength training; and to propose a new Telehealth WTCB exercise for improving neuromuscular functions of old adults with spinal cord injury (SCI) and disability. With reference to neuromuscular functions, WTC intervention may have positive effects on simple reaction time, range of motion at the shoulder and trunk, static and dynamic sitting balance, handgrip strength, vagal activity, and sympathetic activity among older adults with disability. The developed WTCB intervention is a feasible and safe exercise which combines the mind-body exercise and strength conditioning into one exercise which possesses aerobic, stretching and strength trainings and may facilitate neuromuscular functions of older adults with disability. The proposed Telehealth WTCB 12 forms (TWTCB12) exercise with a “Moving Shadow” method in the telehealth may enable the learner to superimpose learner’s image on an expert’s demonstrating model to enhance the learning and practice effects. Since wheelchair users will learn and practice TWTCB12 movements in a seated position or sitting on a wheelchair the “Moving shadow” method on Zoom would provide an ideal telehealth learning and practice environment for the wheelchair users to learn and practice TWTCB12 exercise from home more feasible and user friendly.

## Introduction

There are approximately 65 million people worldwide who have mobility limitations and use wheelchairs as a result of disabling injury or illness (Gurwitz et al., 2021). These individuals with mobility limitations often live a sedentary lifestyle and consequently have issues with cardiovascular, neuromusculoskeletal and bariatric health such as high BMI, and increased body fat percentage (Nooijen et al., 2016). Those compound factors may put older wheelchair users at higher risk of cardiovascular disease, neuromuscular and skeleton disorders which result in lower quality of life (QOL) among them (Jordaan et al., 2017) and would even increase their risk of chronic disease or premature death (Nooijen et al., 2016).

Older adults who use manual wheelchair experience major limitations in their functional activities (Kaye et al., 2000), however, the access to health and exercise programs are limited for individuals who need to exercise in a sitting position or wheelchair. Lack of exercise can result in significant adverse health consequences. Specifically, among older wheelchair users, a physically inactive lifestyle is associated with conditions such as cardiovascular disease, osteoporosis, respiratory complications, muscle mass loss and strength declining, pain and depression (Hicks et al., 2003; Lan et al., 2013; Wang et al., 2020a). Both the physical and mental benefits of exercises have been well documented (Freeman and Lawlis, 2001; Ko et al., 2006; Haskell et al., 2007; van den Berg-Emons et al., 2011; Nguyen and Kruse, 2012). Hence, a health promotion program or exercise focusing on neuromuscular functions is needed for older individuals who use manual wheelchairs or assistive devices as their primary method of mobility.

Tai Chi, a mind-body harmony exercise, consists of a series of graceful movements with deep and slow diaphragmatic breathing performed while standing (Freeman and Lawlis, 2001; Wang et al., 2004). Tai Chi exercise has been shown to have both physical and psychosocial benefits for the general population (Wang et al., 2004; Nguyen and Kruse, 2012; Wayne et al., 2015; Lu et al., 2016; Varghese et al., 2016). Wang et al. (2017) proposed that Tai Chi was a healing movement combining Qi (vital energy), breathing, and stretching techniques. Physically, the slow, graceful movements allow the body to move with less tension and the lungs to be more relaxed, therefore increasing the intake of oxygen (Wang et al., 2016). Mentally, practicing Tai Chi helps decrease stress and anxiety (Abbott and Lavretsky, 2013; Jiang et al., 2016). However, the studies of WTC for individuals with SCI and disability are limited and there are a few Wheelchair or Sitting Tai Chi studies focused on neuromuscular functions among individuals with SCI and disability (Tsang et al., 2015; Qi et al., 2018, 2020; Wang et al., 2016, 2020a).

The purposes of this perspective article were to summarize Wheelchair (Seated) Tai Chi studies related to neuromuscular functions of older adults with disability, to describe the development of WTCB exercise—a concept to combine mind-body exercise with strength training, and to propose a new Telehealth WTCB exercise for improving neuromuscular functions of old adults with SCI and disability.

## Research of Wheelchair Tai Chi or Seated Tai Chi Related to Neuromuscular Functions Among Older Adults With Disability

From the currently available literature, there are a few studies addressing the effects of WTC on the neuromuscular functions among individuals with SCI and disability. Tsang et al. (2015) administrated a sitting Tai Chi intervention to determine its effects on muscle strength, and balance control among individuals with SCI. Eleven participants were engaged in the intervention (90 min/session, 2 times/week for 12 weeks) and eight participants served as controls. The results shown that based on within group comparisons, the sitting Tai Chi group achieved significant improvements in their simple reaction time (*p* = 0.042); in the dynamic sitting balance test: maximum excursion (*p* = 0.016); and directional control (*p* = 0.025) in the limits of stability test after training. Furthermore, they reported that in the sequential weight shifting test: significantly improved their total time to sequentially hit the 12 targets (*p* = 0.035); and significant improvement in handgrip strength (*p* = 0.049) and concluded that 12 weeks of sitting Tai Chi training could improve the dynamic sitting balance and handgrip strength the individuals with SCI (Tsang et al., 2015).

Qi et al. (2018) assessed the effects of wheelchair Tai Chi (WTC) intervention on balance control and QOL among 40 individuals with SCI survivors who were randomly divided into WCTC and control groups. The WCTC group participated in 30-min sessions, 2 sessions/day, and 5 days/week for 6 weeks, while the control group only received the normal rehabilitation intervention. The results shown that the static sitting balance, left handgrip strength, and the psychological domain of QOL improved significantly in the WCTC group (time by group interaction, *p* < 0.05) with comparison to the control group. Therefore, the WCTC exercise may be a feasible, safe, and effective exercise for SCI survivors. Furthermore, Qi et al. (2020) investigated the immediate effect of WTC 16 forms on autonomic nervous modulation among 20 patients with thoracic SCI. The heart rate variability (HRV) of patients at 5 min before and after five consecutive sets of WCTC16 were measured by Equivital-life-monitoring system. The analysis of HRV in the time domain consisted of RR intervals, the standard deviation of all normal R-R intervals (SDNN), and the root mean square of the differences between adjacent NN intervals (RMSSD). The analysis of HRV in the frequency domain consisted of total power (TP) divided into very-low frequency area (VLFP), low-frequency area (LFP), and high-frequency area (HFP) eLF/HF ratio as well as the normalized unit of LFP (LFPnu) and HFP (HFPnu) reflected the sympathovagal balance. Qi et al. (2020) summarized that there was no significant difference in RR interval, SDNN, RMSSD, TP, HEP, VLFP, and LFP of SCI patients before and after WCTC16 exercise (*p* > 0.05). However, LFPnu and HF peak decreased significantly, while HFPnu and LF/HF increased significantly in SCI patients right after WCTC16 exercise (*p* < 0.001). Qi et al. (2020) concluded that the immediate effect of the WCTC16 can improve vagal activity and decrease sympathetic activity, and patients with chronic complete thoracic SCI may achieve the balanced sympathovagal tone.

Our team developed WTC 10 forms in 2008 based on the traditional Tai Chi (Wang et al., 2015). In our pilot study, a 12-week WTC 10 Form (WTC10) intervention was conducted to examine the effect of this WTC10 intervention on selected physical and mental health variables among elderly with disability (Wang et al., 2016). Thirteen in the WTC10 intervention group and 15 in the control group completed the study. The intervention group practiced WTC10 twice a week and 1 h for each session for 12 weeks. The control group only carried on their routine activities of daily living. The outcome measures were: HR and blood pressure, range of motion of the dominant side upper extremity and trunk, and Pain from Self-Efficacy Questionnaire (PSEQ), Physical Activity Scale from Individuals with Physical Disabilities and physical and mental health from SF-36v2 health survey. The results of this pilot study demonstrated that a 12-week WTC intervention had significant effects on blood pressure, range of motion at the shoulder and trunk, physical activity, and mental health among the elderly with disability (Wang et al., 2016).

There are a limited number of studies to address the effect of WTC on the neuromuscular functions among older adults with disability, and the possible improvements related to neuromuscular functions resulted from WTC interventions may be summarized as follows. Tsang et al. (2015) concluded that WTC exercise could improve simple reaction time, dynamic balance, handgrip strength. Qi et al. (2018, 2020) summarized that WTC may improve static sitting balance and vagal activity and sympathetic activity. Wang et al. (2016) reported that WTC may have positive effect on the range of motion at the shoulder and trunk and physical activity ability. However, the current WTC or seated Tai Chi interventions have not included the direct strength training of the upper extremities (Tsang et al., 2015; Shem et al., 2016; Wang et al., 2016; Qi et al., 2018).

## Wheelchair Tai Chi Ball Development and a Pilot Study

Strength training is a crucial exercise for individuals with mobility limitations. Specifically, within several years of mobility limitations, the average cross-sectional area of sub-lesional skeletal muscle, specific to SCI, and peripheral nerve injury decreases by as much as 18–46% progressively (Yarrow et al., 2014). The lack of physical activity and strength training resulted in negative effects on the muscular, skeletal and cardiovascular systems that might lead to secondary complications or death for individuals with mobility limitations (Davis and Shephard, 1990; Cragg et al., 2015). Strength training not only can decrease muscle mass loss, facilitate rapid gains in volitional function and strength (Jayaraman et al., 2013), but also can provide a wide range of benefits imperative to overall health for individuals with mobility limitation (Bochkezanian et al., 2015).

**FIGURE 1:**
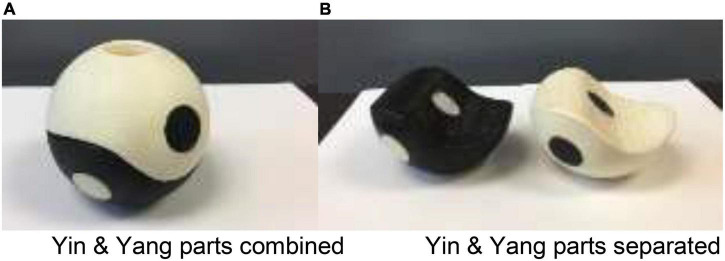
**(A)** Tai Chi Ball is combined with poly-magnets. **(B)** Tai Chi Ball is separated by twisting it.

The rationale for developing WTCB exercise is to take multiple components (physical, mental, and strength conditioning) into consideration. Features of this newly designed ball are: (1) The ball provides resistance for upper extremity strength training; (2) it comes in three sizes (4″, 5″, or 6″) and different weights (1–6 lbs by filling with the weights of 6 mm BB balls) to match individual physical and health conditions (Figures 1A,B); and (3) it can be separated into Yin-Yang parts by twisting it thereby equally distributing the weights to left and right hands or combined (with poly-magnets) as a whole in one hand for weight shifting and strength training. Therefore, the use of the Ball during Tai Chi practice may match the traditional Tai Chi movements well, at the same time may facilitate strength training and neuromuscular functions for older adults with disability. The University of Texas at Tyler in United States has applied the patent for this Tai Chi Ball and the patent application has been approved by US Patent and Trademark Office (Patent No. 10765905) (Wang et al., 2020b).

Based on our previously developed WTC 10 forms which took Yin-Yang Balance, Coordination of Breathing, and Movement Dimensions into consideration (Wang et al., 2015), we developed Wheelchair Tai Chi Ball 12 (WTCB12) forms (Wang et al., 2020a). These 12 forms are (1) Commencing Form (Ball-Separated); (2) Part Wild Horse’s Mane, (Ball-Separated); (3) Brush Knee and Push Forward, (Ball-Separated); (4) Curling the Arms, (Ball-Separated); (5) Left Grasp the Peacock’s Tail (Ball-Together, Ball Transfers from Left to Right Hand); (6) Right Grasp the Peacock’s Tail (Ball-Together, Ball Transfers from Right to Left Hand); (7) Wave Hands Like Clouds, (Ball-Together and Leading Arm with Ball); (8) White Crane Spreads Its Wing (Ball-Together, Transfer Ball to Right Hand); (9) Open and Close hands, (Ball-Separated); (10) Appear Closed (Ball-Separated); (11) Crossing Hands (Ball-Separated); (12) Closing Form (Ball-Separated). Tai Chi is rated as a moderate-intensity exercise integrating graceful physical movements, breathing training, and mindful awareness during practice in standing posture (Wayne et al., 2013). When Tai Chi is performed in a seated position the exercise intensity may decrease since not much or no leg movements are involved for wheelchair users. To add the Tai Chi Ball (weight training) to WTC practice would increase exercise intensity. Our pilot data showed that when practicing WTC with and without the Tai Chi Ball for 20 min, the oxygen consumption (VO2) was 20% higher (*p* < 0.01) and the heart rate (HR) was 5% higher (*p* < 0.01) while carrying the Ball than while not carrying the Ball.

In our pilot study, we examined a 12-week WTCB12 intervention on physical and mental health and function abilities among elderly with physical disability (Wang et al., 2020a). Twenty-six elderly persons participated in the study, nine WTCB group participants and 10 control group participants completed the study. The WTCB group practiced WTCB12 twice/week, 1 h each time for 12 weeks. The control group did their daily routine. The outcomes measures were: PSEQ, physical and mental health from SF-36v2, HR, blood pressure, range of motion, and muscle strength of the dominant arm at the shoulder, elbow and wrist joints. The results between groups demonstrated that the WTCB12 intervention may have significant effects on: (1) PSEQ in the activities of daily living; (2) general physical and mental health; and (3) maintaining the upper extremity muscle strength for the activities of daily living. From observations of the upper arm muscle strength data in Table 1, we found that the muscle strengths at the shoulder, elbow and wrist joints of the WTCB group remain about the same between the pre-test and post-test, but those of the control group decreased significantly in the post-test (Wang et al., 2020a).

**TABLE 1:** Means and SDs of the muscle strength of the upper extremity between the WTCB group and control group.

Variable	WTCB group		Control group	
		
In pounds	Pre-test (SD)	Post-test (SD)	Pre-test (SD)	Post-test (SD)
**Shoulder**				
Flexion	14.67 (3.45)	14.12 (4.75)	14.06 (4.52)	10.15 (5.91)
Extension	19.80 (5.79)	19.61 (7.04)*	16.18 (5.06)	13.28 (5.90)*
Abduction	16.08 (4.87)	14.78 (4.11)*	12.22 (4.15)	9.02 (6.09)*
Adduction	21.65 (6.36)	21.06 (5.78)*	20.14 (5.76)	16.08 (4.42)*
**Elbow**				
Flexion	16.07 (7.59)	16.76 (7.01)*	11.77 (3.40)	9.91 (4.42)*
Extension	16.24 (6.69)	15.18 (5.98)*	13.77 (6.27)	10.19 (2.80)*
**Wrist**				
Flexion	11.54 (5.12)	11.33 (5.33)*	8.81 (3.34)	6.36 (2.30)*
Extension	11.52 (5.93)	10.38 (5.16)*	9.32 (2.65)	5.83 (1.45)*

*N_1_ = 9 in WTCB group and N_2_ = 10 in control group.*

*Significance between groups in the post-test test at the p < 0.05 level.

## Discussion

The results of the upper extremity muscle strengths in the WTCB study might indicate that the WTCB intervention as a combination of both mind-body exercise and strength training may help to maintain upper extremity muscle strengths of the elderly (average 84 years old) with disability. The term “use it or lose it” may well reflect on the strength training in the elderly. Numerous studies have demonstrated that strength training can improve or maintain muscle mass and strength in older adults (Guizelini et al., 2018; Bennie et al., 2019). Furthermore, WTCB exercise is accessible, safe and inexpensive. It is a type of client self-initiated rehabilitation program and could be used in regions where there is a prevalent shortage of resources for hospital- and community-based rehabilitation (Bethge et al., 2014; Zheng et al., 2018).

The key innovation for the WTCB exercise lies in (1) WTCB combines the mind-body exercise and strength conditioning into one exercise which possesses aerobic, stretching and strength training; and (2) the Tai Chi Ball can be split into Yin-Yang parts or combined into one during WTCB exercise so that the use of the Tai Chi Ball during Tai Chi practice may match the traditional Tai Chi movements well, at the same time may facilitate upper extremity strength training and neuromuscular functions. The weight of the ball can be customized for the user, based on the participant’s upper arm strength and physical condition. To our limited knowledge, the WTCB intervention may be the first attempt to combine mind-body and strength training into one exercise and the data of our pilot study showed promising results that the WTCB exercise had positive effects on self-efficacy pain management, general physical health, and maintain upper extremity muscle strength and was a feasible exercise for elderly with disability (Wang et al., 2020a).

**FIGURE 2:**
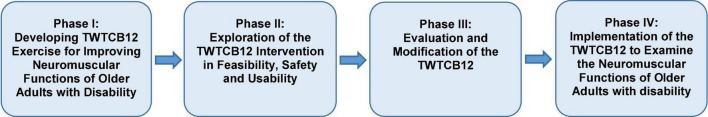
Medical Research Council (MRC) framework for TWTCB12 intervention in four phases for improvement of neuromuscular functions for older adults with disability modified conceptual framework based on the model of Craig et al. (2013).

There are several factors limit wheelchair users’ access to exercise facilities such as transportation to gyms or clinics, and lack of home exercise equipment (Meyers et al., 2002; Cardinal and Spaziani, 2003). Many individuals with mobility limitations do not leave home because they are disabled, and the pandemic situation significantly decreased wheelchair users’ daily activities and their engagement in physical activity and exercise. In recent years, telehealth and virtual rehabilitation become feasible and practical (Escalante-Gonzalbo et al., 2021; Gajarawala and Pelkowski, 2021). Therefore, we propose to add telehealth concept to WTCB12 exercise—to convert WTCB to Telehealth Wheelchair Tai Chi Ball (TWTCB12) and use a “Moving Shadow” method in telehealth that enables the learner to superimpose her/his image on an expert’s demonstrating model to enhance the learning and practice effects. In order for the designed TWTCB12 intervention to improve the neuromuscular functions of older adults, the conceptual framework of the process of development-exploration-evaluation-implementation in four different phases is presented in Figure 2, a modified model of Craig et al. (2013) in order to determine the effects of the TWTCB12 on neuromuscular functions for older adults with disability.

Our *working hypothesis* is that applying the concepts of telehealth and “Moving Shadow” method in TWTCB intervention will make the intervention safe, feasible and user friendly for older wheelchair users with disability. The “Moving shadow” for TWTCB12 intervention demonstrating model is generated by three of the software: Zoom (providing a platform for TWTCB12), ManyCam (adding animation background as the moving shadow for expert’s demonstrating), and OBS Studio (making the participant-self half transparent for superimposing on the demo image). The advantages of this “Moving Shadow” telehealth (online) methods are: (1) Tai Chi is a slow movement and the mirror shadow movements is performed by the Tai Chi master so that it is easy for learner to learn TWTCB12following the shadow by superimposing her/him onto the expert’s image; and (2) The “Moving shadow” method on Zoom would provide an ideal telehealth environment for the wheelchair users to learn and practice WTCB12 exercise from home more feasible and user friendly.

We will conduct a pilot study of TWTCB12 Intervention to examine the neuromusculoskeletal functions of older adults with disability. The outcome measures in this pilot study would be the proprioception and motor coordination (e.g., eye-hand coordination, bilateral coordination, etc.), postural sway, spinal reflex excitability, central nervous system (CNS) responses (e.g., EEG, fMRI, TMS, fNIRs, or reflexes, etc.), muscle size or volume, muscle fiber typing and medical imaging methods (e.g., MRI, CT or ultrasound) in additional the outcome measures in aforementioned literature review. In order to meet the physical conditions of the individuals with limited mobility or SCI, three different weight levels (1–2 lbs; 3–4 lbs; 5–6 lbs) of the Tai Chi Ball are used to meet the physical conditions. Furthermore, we will develop a short form such as “Telehealth Wheelchair Tai Chi Ball 6 Forms (TWTCB6)” which included the TWTCB12 forms 1–3 and forms 10–12 for those who cannot learn and practice the full set of TWTCB12 due to very limited mobility or physical function. Meanwhile, we may also first conduct the TWTCB6 exercise without a Ball for individuals with more limited mobility based on a baseline physical condition evaluation such as the range of motion and muscle strength of the upper extremities. After the participants get stronger, we may engage them in TWTCB12 exercise. We anticipate that the TWTCB12 exercise will be an effective and feasible intervention for the improvement of neuromusculoskeletal functions for older adults with disability.

## Data Availability Statement

The original contributions presented in this study are included in the article/supplementary material, further inquiries can be directed to the corresponding author.

## Author Contributions

TL wrote the outline and draft of the manuscript. X-WK made the literature search and wrote a brief summary. YW guided and revised the first version, the second version and the final version. All authors contributed to the article and approved the submitted version.

## Conflict of Interest

The authors declare that the research was conducted in the absence of any commercial or financial relationships that could be construed as a potential conflict of interest.

## Publisher’s Note

All claims expressed in this article are solely those of the authors and do not necessarily represent those of their affiliated organizations, or those of the publisher, the editors and the reviewers. Any product that may be evaluated in this article, or claim that may be made by its manufacturer, is not guaranteed or endorsed by the publisher.
